# Image Findings of Acute to Subacute Craniocervical Arterial Dissection on Magnetic Resonance Vessel Wall Imaging: A Systematic Review and Proportion Meta-Analysis

**DOI:** 10.3389/fneur.2021.586735

**Published:** 2021-04-07

**Authors:** Se Jin Cho, Byung Se Choi, Yun Jung Bae, Sung Hyun Baik, Leonard Sunwoo, Jae Hyoung Kim

**Affiliations:** Department of Radiology, Seoul National University College of Medicine, Seoul National University Bundang Hospital, Seongnam, South Korea

**Keywords:** arteries, dissection, magnetic resonance imaging, systematic review, meta-analysis

## Abstract

**Background and Purpose:** This systematic review and meta-analysis aimed to evaluate the pooled proportion of image findings of acute to subacute craniocervical arterial dissection (AD) direct signs on magnetic resonance vessel wall imaging (MR-VWI) and to identify factors responsible for the heterogeneity across the included studies.

**Methods:** A systematic literature search in the Ovid-MEDLINE and EMBASE databases was performed for studies published on the relevant topic before April 14, 2020. Pooled sensitivity and specificity values and their 95% confidence intervals (CIs) were calculated using bivariate random-effects modeling. Meta-regression analyses were also performed to determine factors influencing heterogeneity.

**Results:** Eleven articles with data for 209 patients with acute to subacute craniocervical AD who underwent MR-VWI were included in this systematic review and meta-analysis. The most common findings on MR-VWI were wall hematoma (84%; 95% CI, 71%−92%), abnormal enhancement (72%; 95% CI, 49%−88%), aneurysmal dilatation (71%, 95% CI, 53%−84%), and intimal flap or double lumen signs (49%; 95% CI, 29%−71%). Among the potential covariates of heterogeneity, the presence of contrast-enhanced T1-weighted imaging (CE-T1WI) within the MR-VWI sequence combination significantly affected the pooled proportion of the intimal flap or double lumen signs.

**Conclusion:** Wall hematoma and intimal flap or double lumen signs were the most common and least common direct sign image findings, respectively, on MR-VWI in patients with acute to subacute craniocervical AD. Furthermore, the absence of CE-T1WI in MR-VWI protocol was the cause of heterogeneity for the detection of the intimal flap or double lumen signs. This data may help improve MR-VWI interpretation and enhance the understanding of the radiologic diagnosis of craniocervical AD.

## Introduction

Conventionally, digital subtraction angiography (DSA) had been the method of choice for diagnosing craniocervical arterial dissection (AD) using findings such as double lumen and pearl and string signs (1, 2). Due to the invasiveness of DSA, magnetic resonance angiography (MRA) emerged as a non-invasive alternative to the luminal evaluation of craniocervical AD (3). Conventional MRA can provide information about geometric vascular changes, hemodynamic alterations, and collateral blood vessel development, but these are indirect signs, and there are inevitable limitations for the depiction and evaluation of arterial wall pathology (1, 2). Owing to its non-invasiveness and superior performance compared to luminal angiography, high-resolution magnetic resonance vessel wall imaging (MR-VWI) is now widely used for the diagnosis of AD (2, 4–6).

However, despite improving the diagnosis of AD considerably, MR-VWI remains a subject of active ongoing research in terms of aspects such as sequence combinations, spatial resolution, and acquisition techniques (2, 4–6). Correspondingly, studies reporting image findings of direct signs of vascular wall pathology have focused on positive findings on MR-VWI in patients with acute or subacute craniocervical AD (4, 7–15). With this in mind, the recognition of direct signs of MR-VWI would enhance a comprehensive understanding of the radiologic diagnosis of craniocervical AD. Furthermore, the factors responsible for the heterogeneity in techniques, protocols, and image findings of direct signs across studies need to be identified and cross-validated. In this context, this systematic review and meta-analysis aimed to evaluate the pooled proportion of image findings of direct signs of craniocervical AD on MR-VWI and to identify factors responsible for heterogeneity across the included studies. To the best of our knowledge, this is the first systematic review and meta-analysis that pools image findings on MR-VWI in patients with AD.

## Materials and Methods

This study was performed according to the Preferred Reporting Items for Systematic Reviews and Meta-Analyses (PRISMA) guidelines (16).

### Literature Search

A search of the MEDLINE and EMBASE databases was performed to find original literature reporting image findings of direct signs on MR-VWI for patients with acute to subacute craniocervical AD. The following search terms were used: [(Magnetic resonance vessel wall imaging) OR (MR^*^ vessel wall imaging) OR (vessel wall imaging) OR (vessel wall MR^*^) OR (high-resolution MR^*^) OR (high-resolution magnetic resonance)] AND [(dissect^*^)]. No initial search date was set, and the literature search was updated until April 14, 2020. The search was limited to publications in English. The bibliographies of relevant articles were searched to identify other relevant articles.

### Inclusion Criteria

Studies satisfying the following criteria were included: (1) involved patients with acute to subacute craniocervical AD; (2) used MR-VWI as the index test; and (4) contained sufficient information regarding the proportion of image findings of direct signs in acute to subacute craniocervical AD on MR-VWI.

### Exclusion Criteria

The following articles or article subset types were excluded: (1) case reports or series including fewer than five patients; (2) letters, editorials, conference abstracts, systematic reviews or meta-analyses, consensus statements, guidelines, and review articles; (4) articles not focusing on the current topic; (5) articles with, or with suspicion of, overlapping populations; and (6) articles containing insufficient information on the proportion of image findings of direct signs of acute to subacute craniocervical AD on MR-VWI.

Two radiologists, S.J.C and B.S.C, with 7 and 19 years of experience in neuroimaging, respectively, independently performed the literature search and selection.

### Data Extraction

The following data were extracted using standardized forms according to the PRISMA guidelines (16): (1) characteristics and demographic data of the included studies: author names, year of publication, institution, country of origin, period of patient recruitment, study design (prospective vs. retrospective), patient number, male-to-female ratio, mean age of enrolled patients (adult or not), number of patients with acute to subacute craniocervical AD, reference standard, clinical diagnostic criteria for AD, mean interval days from symptom to MR-VWI and their ranges, AD location (intracranial vs. extracranial), and mechanism of the AD (spontaneous vs. traumatic); (2) patient symptoms and underlying risk factors at initial presentation; (4) MR-VWI characteristics: MRI machine and vendor, magnetic field strength, MRI dimension (2D or 3D), coil type, under-sampling techniques, use of the black blood (BB) technique during image acquisition, MR-VWI sequence combination, repetition time/echo time, field of view, slice thickness, acquisition matrix of T1-weighted imaging (T1WI) as a representative sequence, analytical methods to evaluate MR-VWI (subjective or objective), and the interobserver agreement for diagnosis; and (5) the proportion of image findings of direct signs on MR-VWI, luminal stenosis on angiography, and presence of infarction.

AD was defined as acute stage if it was detected within 3 days of symptom onset, subacute stage 4–60 days, and chronic stage after 60 days (17).

### Quality Assessment

The methodological quality of the included studies was evaluated using tailored questionnaires and the Quality Assessment of Diagnostic Accuracy Studies-2 (QUADAS-2) criteria (18). Two reviewers (S.J.C and B.S.C) independently performed data extraction and quality assessment.

### Data Synthesis and Analyses

The primary aim was to describe the demographic data and perform pooled proportion analysis of image findings of direct signs of acute to subacute craniocervical AD on MR-VWI. The secondary aim was to identify factors responsible for the heterogeneity across the included studies by employing meta-regression analysis. Pooled proportions were calculated using an inverse-variance weighting model (19–21). A random-effects meta-analysis of proportions was utilized to calculate the overall proportions. Study heterogeneity was evaluated using the Higgins inconsistency index (*I*^2^), with substantial heterogeneity indicated by an *I*^2^ value >50% (22). In addition, meta-regression analysis was performed for the pooled proportion of image findings to determine the factor for the heterogeneity. All statistical analyses were conducted by one author (S.J.C, with 3 years of experience in conducting systematic reviews and meta-analysis) using the “meta” package of R (version 3.6.3; http://www.r-project.org/).

## Results

### Literature Search

**Figure 1:**
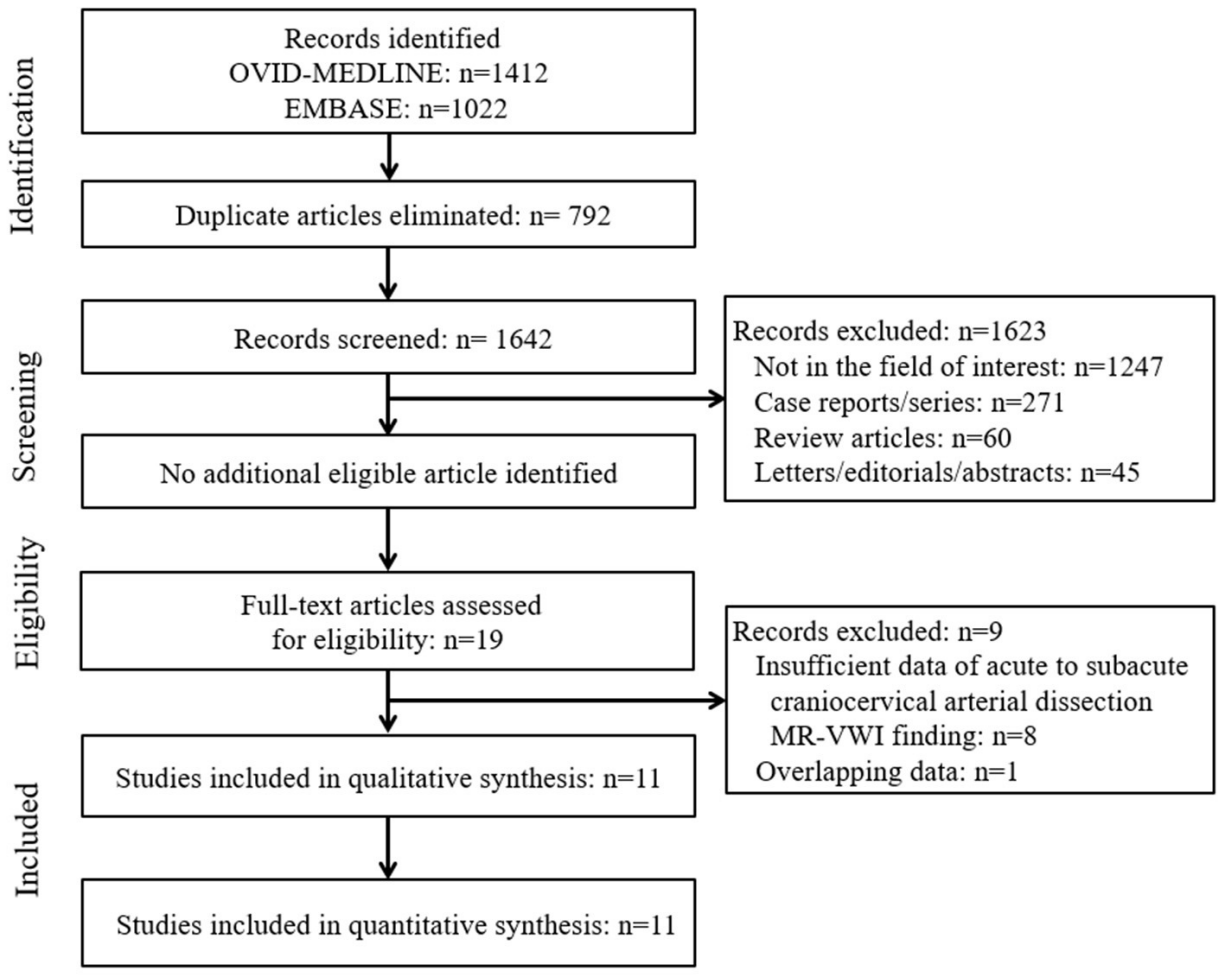
Flow diagram of the study selection process.

The systematic literature search (Figure 1) initially identified 2,434 articles. After removing 792 duplicates, screening of the remaining 1,642 titles and abstracts yielded 19 potentially eligible articles. No additional articles were identified after searching their bibliographies. After full-text reviews of these 19 articles, eight were excluded because they contained insufficient information in terms of the proportion of image findings of acute to subacute craniocervical AD on MR-VWI (2, 5, 6, 23–27) and one because of suspected population overlap (28). Finally, 11 articles were included in the final analysis (4, 7–15, 29).

### Characteristics of the Included Studies

The total number of patients with AD across all studies was 327 (range, 6 to 118 patients). The number of dissected arteries across all studies was 372 (range, 6 to 145) (Table 1). The mean patient age across studies ranged from 40.4 to 55 years. Except for one patient in one study, all studies included adults (10). Eight of the included articles were retrospective in design (4, 7, 8, 11, 13–15, 29), whereas three studies were prospective (9, 10, 12). The reference standard for diagnosing AD was a combination of clinical, radiological, and luminal angiography diagnoses in three studies (12, 14, 29) and of clinical, radiological, luminal angiography, and follow-up MRI in eight studies (4, 7–11, 13, 15). Two of these studies specifically used the spontaneous AD criteria for clinical diagnosis (8, 15), and two used the Strategies Against Stroke Study for Young Adults in Japan criteria (11, 14). The mean intervals from symptom to MR-VWI were shorter than 10 days in six of the included studies (4, 9, 12–15) and equal to or longer than 10 days in four (7, 8, 11, 29); the relevant information was not specified in a remaining article (10). AD was located exclusively in posterior circulation in six studies (4, 8, 9, 13–15) and predominantly in posterior circulation (in more than half the cases) in two studies (11, 29); the locations were variable in the other studies (7, 10, 12). Seven studies only evaluated intracranial arteries (4, 8, 9, 11, 13–15) and three studies evaluated both intracranial and extracranial arteries (7, 10, 29); the relevant information was not specified in a remaining article (12).

**Table 1:** Characteristics of the included studies.

**Source**	**Affiliation**	**Period of patient recruitment**	**Study design**	**Pt. no**.	**AD no**.	**Mean age**	**Male/female**	**Adult**	Reference standard/criteria^*^	**Mean days from Sx. to VWI (range)**	**Location of AD**	**Intracranial vs. extracranial**	**Mechanism of AD**
Coppenrath et al. (12)	Ludwig Maximilian University Munich, Germany	August 2007–June 2010	Pros.	33	44	47	19/14	All	Clinico. + Radio. + Angio./NA	NA (<7)	29 ICA, 15 VBA	NA	All spontaneous
Cuvinciuc et al. (7)	University Hospitals of Geneva, Switzerland	NA	Retro.	14	14	45.1	11/3	All	Clinico. + Radio. + Angio. + FU Radio./NA	12 (1–45)	9 ICA, 5 VBA	Both	10 spontaneous, 4 traumatic
Han et al. (4)	Ajou University Medical Center, Republic of Korea	March 2012–October 2013	Retro.	33	35	51.1	28/3	All	Clinico. + Radio. + Angio. + FU Radio./NA	2.5 (1–7)	1 BA, 9 PICA, 25 VA	Intracranial	NA
Jung et al. (11)	Asan Medical Center, Republic of Korea	April 2012–February 2015	Retro.	26	28	47	17: 9	All	Clinico. + Radio. + Angio. + FU Radio./SASSY-Japan	10 (1–30)	6 ACA, 4 MCA; 18 VBA	Intracranial	All spontaneous
Kano et al. (15)	Tosei General Hospital, Japan	March 2016–July 2019	Retro.	6	6	44.8	4: 2	All	Clinico. + Radio. + Angio. + FU Radio./SCADS	5 (2–14)	All PICA	Intracranial	All spontaneous
Li et al. (10)	Beijing Tiantan Hospital, China	June 2012–September 2014	Pros.	24	24	45	21: 3	Almost^‡^	Clinico. + Radio. + Angio. + FU Radio./NA	NA (<14)	11 ICA, 3 MCA, 10 VBA	Both	All spontaneous
Natori et al. (9)	Iwate Medical University, Japan	April 2011–March 2013	Pros.	16	16	55	NA	All	Clinico. + Radio. + Angio. + FU Radio./NA	9.5 (0–37)	All VBA	Intracranial	All spontaneous
Ogawa et al. (13)	Nagoya City University Graduate School of Medical Sciences, Japan	January 2015–February 2017	Retro.	12	14	52	9: 3	All	Clinico. + Radio. + Angio. + FU Radio./NA	7.3 (1–21)	All VBA	Intracranial	NA
Sakurai et al. (8)	Nagoya City University Graduate School of Medical Sciences, Japan	November 2009–April 2011	Retro.	15^†^	17^†^	51^†^	NA^†^	All	Clinico. + Radio. + Angio. + FU Radio./SCADS	22 (2–57)	All VBA	Intracranial	All spontaneous
Wu et al. (29)	Xuanwu Hospital, China	September 2013–September 2018	Retro.	145	145	40.4	95, 50	All	Clinico. + Radio. + Angio./NA	12 (6–20)	77 VBA, 66 ICA, 1 PCA, 21 multiple	Both	NA
Yun et al. (14)	Busan Paik Hospital, Republic of Korea	May 2013–August 2017	Retro.	27	29	52.5	15, 12	All	Clinico. + Radio. + Angio./SASSY-Japan	4 (0–60)	All VBA	Intracranial	All spontaneous

*ACA, anterior cerebral artery; BA, basilar artery; AD, arterial dissection; MCA, middle cerebral artery; ICA, internal cerebral artery; NA, not available; no., number; Pt., patient, PICA, posterior inferior cerebellar artery; Pros, prospective; Retro, retrospective; Sx., symptom; VA, vertebral artery; VBA, vertebrobasilar artery; VWI, vessel wall imaging; SCADS, Spontaneous coronary artery dissection criteria; SASSY-Japan, Strategies Against Stroke Study for Young Adults in Japan*.

* *Combination of reference standard: Clinico*.

+ Radio. + Angio+ FU Radio means consensus diagnosis by clinical, radiologic (MRI), luminal angiographic, and follow-up radiology examinations;

† we extracted only acute- to subacute-staged AD of 15 patients' data from 20 AD of 18 patients (the mean age was that of 18 patients, and the male/female ratio of 18 patients were 14:4);

‡ *except only a 1-year-old female*.

All but three of the studies included spontaneous AD (8–12, 14, 15); one study partly included traumatic AD (7), and three studies had unclear information in this regard (4, 13, 29). The pooled proportion of symptoms at presentation and the underlying risk factors of patients are presented in Table 2. The most common symptoms were pain [44%; confidence interval (CI), 32%−57%], motor weakness (33%; 95% CI, 22%−46%), and dizziness (21%; 95% CI, 8%−45%). Common underlying risk factors were smoking (31%; 95% CI, 22%−43%), hypertension (29%; 95% CI, 19%−43%), and dyslipidemia (20%; 95% CI, 14%−26%).

**Table 2:** Pooled proportion of symptoms at presentation and the risk factors of patients with arterial dissection.

**Source**	**Symptom**							**Risk factor**				
	Pain^*^	**Motor weakness^†^**	**Dizziness**	**Sensory change**	**Horner syndrome**	**Mental change**	**Others^‡^**	**HTN**	**Smoking**	**Dyslipidemia**	**DM**	**Others^§^**
Coppenrath et al. (12)	10	17	0	4	4	0	3	12	18	10	0	5
Cuvinciuc et al. (7)	3	7	1	1	7	1	2	NA	NA	NA	NA	NA
Han et al. (4)	16	5	20	6	0	0	12	14	17	5	6	2
Jung et al. (11)	13	10	1	2	0	0	0	13	12	9	1	0
Kano et al. (15)	4	0	6	0	0	0	4	NA	NA	NA	NA	NA
Li et al. (10)	7	NA	NA	NA	NA	NA	NA	13	10	0	6	0
Natori et al. (9)	NA	NA	NA	NA	NA	NA	NA	4	0	1	2	0
Ogawa et al. (13)	8	5	3	0	0	0	2	NA	NA	NA	NA	NA
Sakurai et al. (8)	15	5	1	0	0	1	0	4	3	5	0	1
Wu et al. (29)	NA	NA	NA	NA	NA	NA	NA	17	33	27	NA	57
Yun et al. (14)	10	5	9	0	0	1	0	6	5	4	2	5
**Pooled proportion, % (95% CI)**	44 (32–57)	33 (22–46)	21 (8–45)	11 (7–17)	7 (2–21)	4 (2–9)	15 (6–32)	29 (19–43)	31 (22–43)	20 (14–26)	11 (6–20)	9 (4–22)

CI, confidence interval; DM, diabetes mellitus; HTN, hypertension; NA, not available. Encompassed

* headache and neck pain;

† emiparesis, dysarthria, dysphagia, or Wallenberg or lateral medullary syndrome;

‡ vomiting, nausea, etc.; and

§ *familial history of risk factor, coronary artery disease, etc*.

### MR-VWI-Associated Characteristics of Included Studies

In seven studies, 3-T scanners were used (4, 10–14, 29) and 1.5-T scanner in four (Table 3) (7–9, 15). Eight studies used 3D-MRI (7–11, 13, 15, 29), one used 2D-MRI (12), and one used either 2D- or 3D-MRI (14); the technique used was not specified in one study (4). The included studies used different coil types. Three studies acquired MRI data using under-sampling techniques, all of which were parallel imaging techniques (8, 9, 15). Five studies used the BB technique (4, 8, 10, 12, 13). Regarding MR-VWI sequence combinations, all studies included T1WI-based sequences, whereas the use of contrast enhanced-T1WI (CE-T1WI), T2WI, and proton density imaging was variable. T1WI (as a representative MR-VWI sequence) acquisition parameter details are presented in Table 3. Slice thicknesses <1 mm were used in seven studies (7–10, 13, 15, 29); in two of these, the thickness was <0.6 mm (7, 9). Nine studies used subjective image analysis (4, 7–10, 12, 13, 15, 29), whereas two studies added objective measurement for the image findings (11, 14). Four studies revealed the interobserver agreement in diagnosing AD between two observers, which were all moderate to excellent (kappa range, 0.73–1) (4, 7, 9, 13).

**Table 3:** Characteristics of magnetic resonance vessel wall imaging.

**Source**	**Protocol**							**T1**	**T1WI**	**T1WI**	**T1WI**	**Assessment**		
	**Machine**	**T**	**D**	**Coil**	**Under-sampling^†^**	**BB**	**Sequence combination**	**TR/TE, ms^†^**	**FOV, mm^**2**^^†^**	**Slice thickness, mm**	**Acquisition matrix^†^**	**Methods (reader no.)**	**Interobserver agreement for diagnosis**	**Visual (subj.) assessment**
Coppenrath et al. (12)	Verio, Siemens	3	2	Flexible 4-channel carotid surface coil	NA	Yes	T1WI, CE-T1WI, CE-MRA	800/12	160 × 120	2	240 × 320	Subj. (2)	NA	Vessel wall hematoma, abnormal enhancement, luminal occlusion, and presence of infarction
Cuvinciuc et al. (7)	Espree MRI scanner, Siemens	1.5	3	Standard head and neck coil	NA	No	T1WI (SPACE), CE-MRA,	750/ 2	230 × 230	0.45	256 × 256	Subj. (2)	Kappa 0.82–1	Vessel wall hematoma
Han et al. (4)	Intera Achieva, Philips	3	NA	16-channel neurovascular head coil	NA	Yes	T1WI, T2WI, PD, CE-T1WI, TOF-MRA	1,000/7.9	100 × 100	2	20 × 200	Subj. (2)	Kappa 0.83^*^	Vessel wall hematoma, intimal flap or double lumen, luminal occlusion, aneurysmal dilatation, and presence of infarction
Jung et al. (11)	Achieva, Philips	3	3	8-channel head coil	NA	No	T1WI, T2WI, PD, CE-T1WI, TOF-MRA	695/80	100 × 100	1	512 × 512	Subj. + Obj. (2)	NA	Vessel wall hematoma, intimal flap or double lumen, abnormal enhancement, an extension to other arteries, and aneurysmal dilatation
Kano et al. (15)	MAGNETOM Aera, Siemens	1.5	3	20-channel head and neck coil	GRAPPA factor, 2	No	T1WI, T2WI, TOF-MRA, BPAS	600/78	210 × 210	0.8	256 × 230	Subj. (2)	NA	Vessel wall hematoma, aneurysmal dilatation, and luminal stenosis or occlusion
Li et al. (10)	Achieva TX, Philips	3	3	36-channel neurovascular coil	NA	Yes	T1WI (SNAP), T2^*^WI (MERGE), TOF-MRA	10/4.8	250 × 160	0.8	NA	Subj. (2)	NA^†^	Vessel wall hematoma, luminal stenosis or occlusion, and presence of infarction
Natori et al. (9)	Signa HDxt, GE	1.5	3	8-channel head coil	Parallel imaging factor, 2	No	T1WI (flow-sensitive FSE), T2WI, TOF-MRA, BPAS	500/18	250 × 190	0.5	512 × 512	Subj. (1)	Kappa 0.73	Vessel wall hematoma, intimal flap or double lumen, luminal stenosis, and aneurysmal dilatation
Ogawa et al. (13)	Trillium Oval, Hitachi	3	3	15-channel head coil	NA	Yes	T1WI, CE-T1WI (iso FSE), T2WI, TOF-MRA	487/16	170 × 170	0.9	224 × 204	Subj. (2)	Kappa 0.90–1	Vessel wall hematoma, intimal flap or double lumen, abnormal enhancement, and aneurysmal dilatation
Sakurai et al. (8)	Gyroscan Intera; Philips	1.5	3	SENSE head coil	SENSE factor, 2	Yes	T1WI (VISTA and 2D-BBT1WI), CE-T1WI (3D.-SPGR), TOF-MRA	400/13	180 × 180	0.9	256 × 256	Subj. (2)	NA	Vessel wall hematoma, intimal flap or double lumen, abnormal enhancement, aneurysmal dilatation, and presence of infarction
Wu et al. (29)	MAGNETOM Verio, Siemens	3	3	32-channel head/neck coil	NA	No	T1WI (SPACE), CE-T1WI (SPACE),	900/14	230 × 230	0.6	288 × 384	Subj. (2)	NA^‡^	Vessel wall hematoma, intimal flap or double lumen, luminal stenosis, abnormal enhancement, and presence of infarction^§^
Yun et al. (14)	Achieva, Philips; MAGNETOM Skyra, Siemens	3	2 or 3	8-channel, 64-channel head coil	NA	No	T1WI, PD, CE-T1WI, TOF-MRA	670–100/7.6–8.7	100–170 × 100–170	0.6–2.0	200–256 × 200–256	Subj. + Obj. (NA)	NA	Vessel wall hematoma, intimal flap or double lumen, abnormal enhancement, luminal stenosis or occlusion, aneurysmal dilatation, and presence of infarction

BB, black blood technique; BPAS, basi-parallel anatomical scanning; D, dimension; PD, proton density imaging; FSE, fast spin echo; GRAPPA, Generalized autocalibrating partially parallel acquisition (as a parallel imaging technique); MERGE, multiple echo recombined gradient echo (as a spoiled T2*-weighted sequence); MRA, magnetic resonance angiography; CE, contrast-enhanced; TOF, time-of-flight; NA, not available; SENSE, sensitivity-encoding (as a parallel imaging technique); SNAP, simultaneous non-contrast angiography and intraplaque hemorrhage (as a heavily T1WI); SPGR, spoiled gradient recalled (as a spoiled gradient echo sequence); T, Tesla; TR/TE, repetition time/echo time; (CE)T1WI, (contrast-enhanced) T1-weighted image; T2WI, T2-weighted image; VISTA, volume isotropic turbo spin echo acquisition (as a FSE). NOTE: The MRI parameters presented were that of the representative T1WI sequence;

* 0.66 to 1 for wall hematoma, 0.60 to 0.89 for intimal flap or double lumen sign, 0.81 to 0.88 for aneurysmal dilatation;

† they assessed agreement between the sequences, not between reviewer;

‡ 0.91 for intimal flap or double lumen sign and 0.90 for wall hematoma;

§ *they evaluated only pseudoaneurysm, not outer wall dilatation*.

### Pooled Proportions of Image Findings on MR-VWI

**Figure 2:**
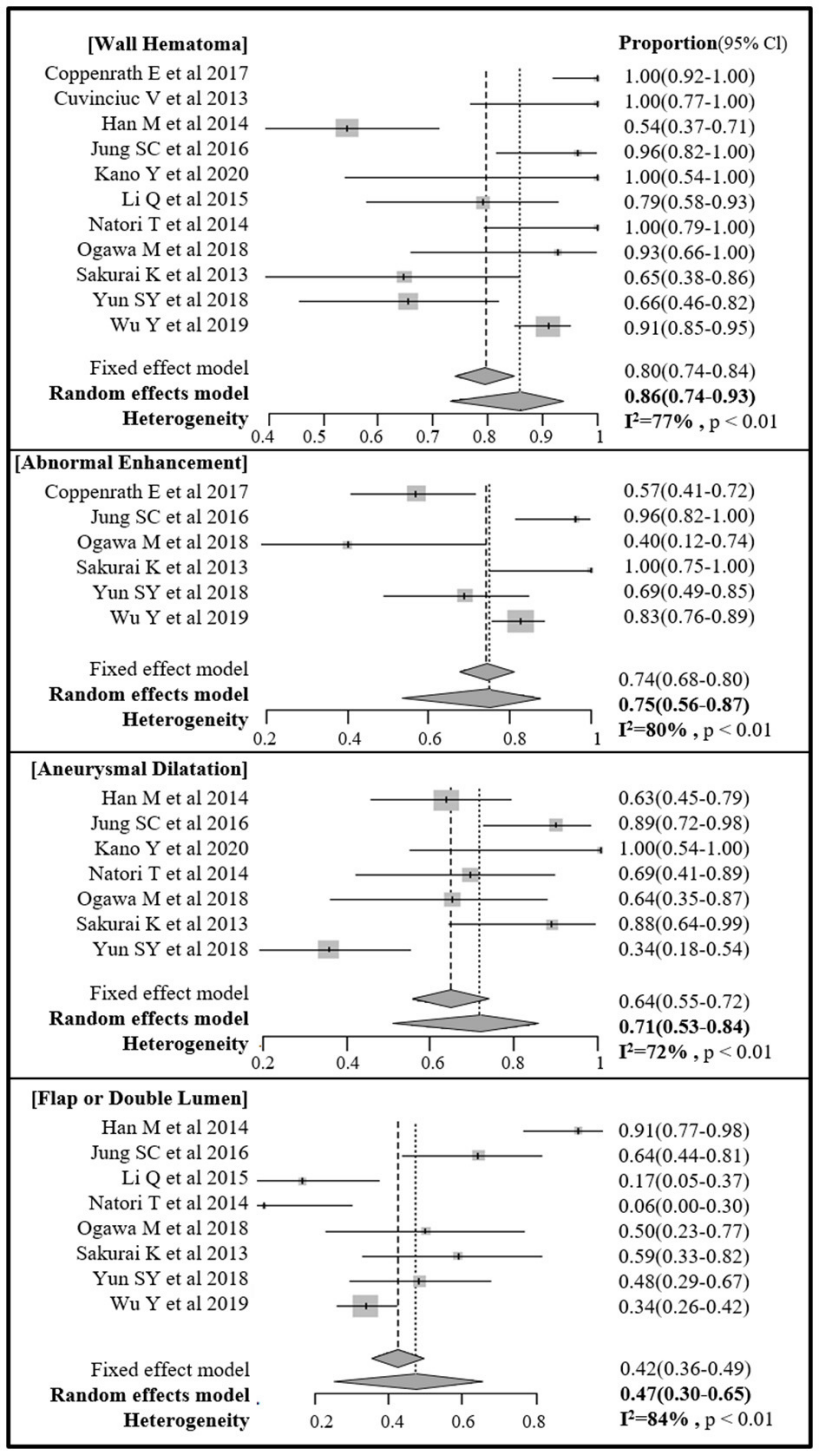
Forest plot of pooled proportion of image findings of direct signs of craniocervical arterial dissection on magnetic resonance (MR) vessel wall imaging.

The pooled proportions of the presence of wall hematoma, abnormal enhancement, aneurysmal dilatation, and intimal flap or double lumen signs on MR-VWI for patients with craniocervical AD were 86% (95% CI, 74%−93%), 75% (95% CI, 56%−87%), 71% (95% CI, 53%−84%), and 47% (95% CI, 30%−65%), respectively (Table 4, Figure 2). Heterogeneity regarding image findings was noted across studies (*I*^2^ = 72%−84%). Pooled proportions of luminal stenosis or occlusion on luminal angiography and the presence of infarction were 76% (95% CI, 55%−89%) and 55% (95% CI, 39%−70%), respectively. Heterogeneity regarding luminal stenosis or occlusion and the presence of infarction across studies were noted (*I*^2^ = 77% and 81%, respectively).

**Table 4:** Pooled proportion of image findings for arterial dissection on magnetic resonance vessel wall imaging.

**Source**	**Image findings of direct signs**				**Luminal stenosis or occlusion on angiography**	**Number of patients with infarction**
	**Wall hematoma**	**Abnormal enhancement**	**Aneurysmal dilatation**	**Intimal flap or double lumen sign**		
Coppenrath et al. (12)	44	25	NA	NA	NA	17
Cuvinciuc et al. (7)	14	NA	NA	NA	NA	NA
Han et al. (4)	19	NA	22	32	17	28
Jung et al. (11)	27	27	25	18	NA	NA
Kano et al. (15)	6	NA	6	NA	5	NA
Li et al. (10)	19	NA	NA	4	19	20
Natori et al. (9)	16	NA	11	1	16	NA
Ogawa et al. (13)	13	4 of 10^*^	9	7	NA	NA
Sakurai et al. (8)	11	13 of 13^*^	15	10	10	4
Wu et al. (29)	132	120	NA	49	145	79
Yun et al. (14)	19	20	10	14	15	13
**Pooled proportion, % (95% CI)**	86 (74–93)	75 (56–87)	71 (53–89)	47 (30–65)	76 (55–89)	55 (39–70)

*CI, confidence interval*.

* *All patients did not undergo contrast-enhanced imaging*.

### Meta-Regression

We used meta-regression analysis to determine the causes of heterogeneity. The covariates evaluated were mean interval from symptom to MR-VWI (<10 vs. ≥10 days), a predominance of posterior circulation in terms of AD location (more than half), magnetic resonance field strength (1.5 T vs. 3 T), performance via the BB technique during image acquisition, presence of CE-T1WI within the MR-VWI sequence combination, repetition time (<500 vs. ≥500 ms), and slice thickness (≥0.6 vs. <0.6 mm). Only the presence of CE-T1WI within the MR-VWI sequence combination was significantly associated with heterogeneity in the proportion of image findings of direct signs on MR-VWI, as it affected the pooled proportion of intimal flap or double lumen signs (*p* < 0.001). All other associations were statistically insignificant.

### Quality Assessment

**Figure 3:**
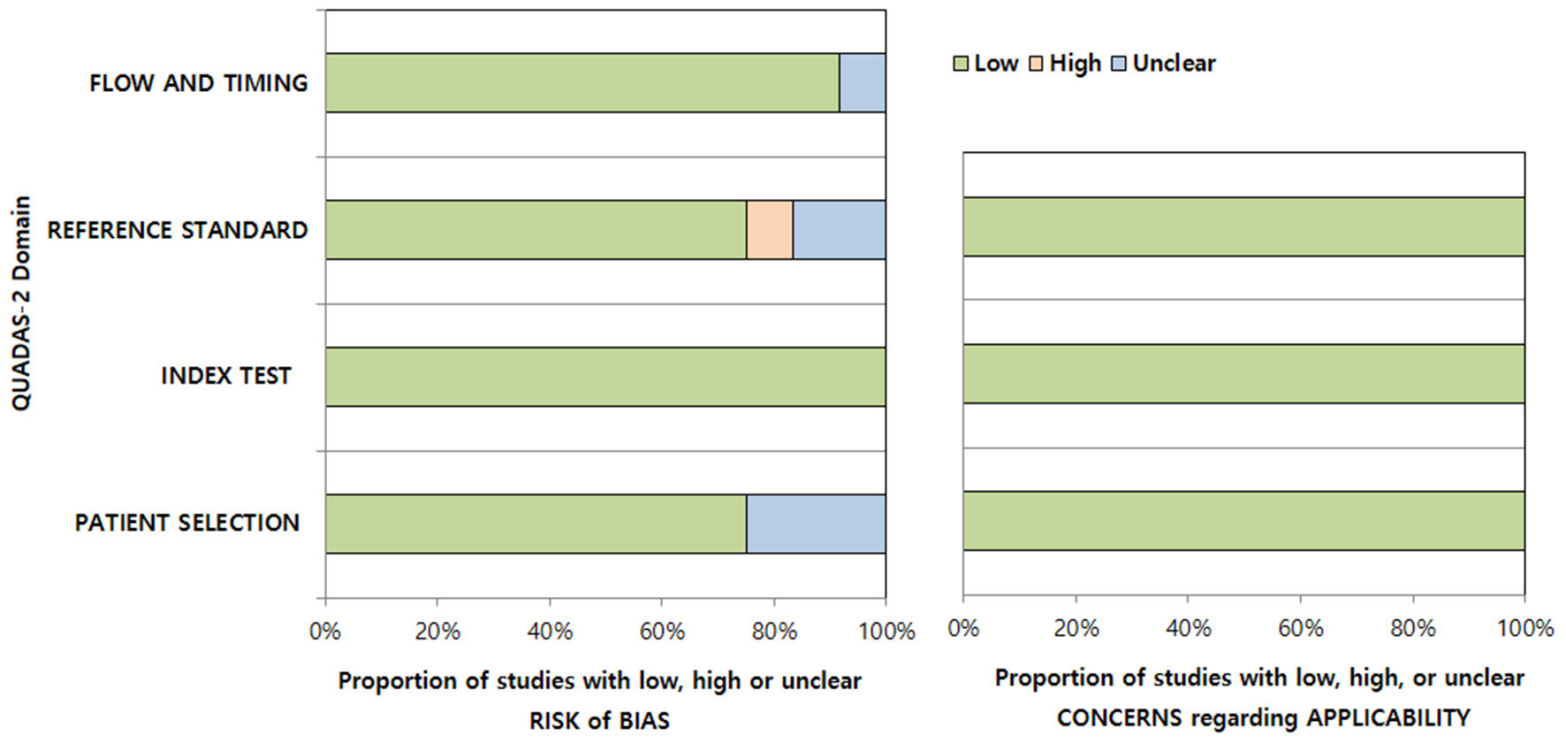
Quality assessment of the included studies according to the Quality Assessment of Diagnostic Accuracy Studies-2 (QUADAS-2) criteria.

The bias risks according to the QUADAS-2 criteria were evaluated (Figure 3). We considered low bias risk in all studies in the index test domain as they all enrolled patients based on clinico-radiologic consensus, despite the absence of surgery considering the in-nature characteristics of diagnosis of this disease. In the patient selection domain, three studies were considered to have an unclear risk of bias due to unclear blinding or non-consecutive patient enrollment (7, 13). In the reference standard domain, one study was considered to have an unclear risk of bias due to the absence of follow-up MRI confirmation of AD (12) and another due to unclear blinding to the index test (11). One study was considered to have a high risk of bias due to both unclear blinding and absence of follow-up MRI confirmation of AD (14). In the flow and timing domain, one study was considered to have an unclear risk of bias due to the time intervals from symptom to MRI being too heterogeneous (1 to 45 days) and a few suspicions of inappropriate patient exclusion (7). All studies were considered to have low applicability in the patient selection, index test, and reference standard domains.

## Discussion

This systematic review and meta-analysis present the pooled proportions of image findings of acute to subacute craniocervical AD on MR-VWI. The most common findings on MR-VWI were wall hematoma, abnormal enhancement, aneurysmal dilatation, and intimal flap or double lumen signs. Among the potential covariates of heterogeneity, the presence of CE-T1WI within the MR-VWI sequence combination caused heterogeneity for the pooled proportion of intimal flap or double lumen signs. The pooled proportion of image findings provides information that can aid MR-VWI interpretation and can be used to enhance the comprehensive understanding of the radiologic diagnosis of craniocervical AD.

Recently, high-resolution MR-VWI has been widely adopted and has reported superior performance to luminal angiography in the diagnosis of AD (2, 4–6). Also, several studies reported image findings of direct signs of dissected vessel wall on MR-VWI (4, 7–15). These findings involve wall hematoma (either in the false lumen or wall layer), outer wall aneurysmal dilatation, abnormal enhancement (either in the wall or flap), and presence of intimal flap and/or double lumen signs. Wall hematoma, the most common finding in craniocervical AD, is predominantly seen as the mural iso- to hyperintensity on T1WI (3/4). However, it can be confused as intraplaque hemorrhage in atherosclerosis, and hypointensity can also be observed on T1WI. The signal intensity on T2WI also contributes to the recognition of the stage of hematoma (2). The reason for abnormal enhancement in patients with AD is not fully understood yet. Inflammation, slow blood flow in the false lumen, and enhancement of the vasa vasorum are possible reasons for enhancement (30, 31). Aneurysmal dilatation of the outer wall can be measured at the perpendicular plane of the diseased vessel, as MR-VWI allows clear discrimination between the outer wall and cerebrospinal fluid (32). Although direct visualization of the intimal flap and/or double lumen signs is the most reliable indicator, their incidence was the least in the current pooled analysis (4).

Although MR-VWI has improved the diagnostic accuracy of AD, it remains a developing field in terms of sequence combinations, spatial resolution, and acquisition techniques (2, 4–6). MR-VWI protocols are inevitably heterologous, giving rise to the necessity for optimization. This meta-analysis also showed heterogeneity in image findings of direct signs of craniocervical AD across studies. In terms of sequence combinations, non-enhanced T1WI, T2WI, proton density, and enhanced T1WI sequences are generally adopted for MR-VWI (3). Han et al. (4) proposed that CE-T1WI was necessary, as they found dissection flaps in almost all cases with CE-T1WI, with substantial agreement among the readers. Correspondingly, this meta-analysis also suggests that the use of CE-T1WI within MR-VWI sequence combinations caused heterogeneity for the detection of the intimal flap or double lumen signs. Although the effects of the other sequences could not be statistically evaluated in this meta-analysis, the combinations of non-enhanced T1WI, T2WI, proton density, and enhanced T1WI sequences are considered essential for evaluation and differential diagnosis of wall hematoma, dissection states, and vessel contours (3). The effectiveness of susceptibility-weighted imaging for detection of wall hematoma and diagnosis of craniocervical AD has also been suggested (24, 25). Therefore, multi-sequential combinations need to be further evaluated in terms of the protocol.

Although significance was not gauged in this meta-regression due to the relatively small number of enrolled studies, there are several factors to be considered for protocol optimization. In two of the included studies, image acquisition slice thickness was more than 1 mm (4, 12). As the craniocervical vascular wall is a thin structure, the recommended slice thickness for MR-VWI is <1 mm; therefore, thicker slices need to be avoided (3). Recently, novel techniques, including the BB (32–36) and under-sampling techniques (37, 38) that improve the resolution and clinical accessibility of MR-VWI are actively being validated. Five of the studies included in this meta-analysis used the BB technique (4, 8, 10, 12, 13); due to suppression of arterial blood and cerebrospinal fluid signals, this technique performs better in intracranial wall evaluation than 3D turbo-spin echo alone (39–42), but there are also some drawbacks, including a reduced signal-to-noise ratio of the vessel wall (32). A recent study evaluated the feasibility of the BB technique in the diagnosis of vertebrobasilar AD (5) and revealed that combining the 3D CE-T1WI and BB techniques reinforced the diagnostic performance of MR-VWI. Various under-sampling techniques, such as parallel imaging techniques, have been developed to simultaneously achieve a reduction of scan time while preserving image quality and scan range (43–45). Currently, parallel imaging techniques such as sensitivity encoding (SENSE) and generalized auto-calibrating partial parallel acquisition are limited in their ability to do this (43, 45) compared to non-uniform sampling techniques such as compressed sensing (CS) or combined CS-SENSE (46). Only three of the studies included in this meta-analysis used under-sampling techniques, and all of these were parallel imaging techniques (8, 9, 15). As the use of under-sampling techniques highly depends on the vendor, they are generally not easy to adopt. Thus, non-uniform sampling (CS or CS-SENSE) for craniocervical AD needs to be further evaluated.

This study had certain limitations. First, the number of included studies and their patient sample sizes were small; therefore, the statistical power of the proportion meta-analysis was low. Second, owing to the inherent scarcity of patients who undergo surgery for craniocervical AD, studies generally enrolled patients based on clinico-radiologic consensus. Third, as AD may present with chronologic and subsequent geometric changes after onset, we only included studies focusing on acute to subacute, and not chronic, AD. Further investigations regarding chronic AD and the corresponding chronologic changes are needed. Fourth, owing to the definition of MR-VWI is established recently, some papers could be missed by our searching process. Fifth, asymptomatic or mild symptomatic patients with AD may be lost in the original studies due to the absence of MR-VWI in the diagnostic process. Finally, we did not perform pooled analysis for objective quantitative measurements, as only a few studies provided the required data.

## Conclusion

Our results indicate that wall hematoma and intimal flap or double lumen signs are the most and least common direct image findings, respectively, on MR-VWI in patients with craniocervical AD. Furthermore, the absence of CE-T1WI in MR-VWI protocol was the cause of heterogeneity for the detection of the intimal flap or double lumen signs.

## Data Availability Statement

The original contributions presented in the study are included in the article/supplementary materials, further inquiries can be directed to the corresponding author/s.

## Author Contributions

SC and BC: conception and design of the study and acquisition and analysis of data. SC, BC, YB, SB, LS, and JK: drafting a significant portion of the manuscript or figures. All authors contributed to the article and approved the submitted version.

## Conflict of Interest

The authors declare that the research was conducted in the absence of any commercial or financial relationships that could be construed as a potential conflict of interest.

## Abbreviations

AD: arterial dissection
BB: black blood
CE-T1WI: contrast enhanced T1-weighted imaging
MRA: magnetic resonance angiography
MR-VWI: magnetic resonance vessel wall imaging.
